# In-depth psychometric evaluation of the PsychQOL questionnaire: factor structure and ROC analysis

**DOI:** 10.3389/fpsyg.2026.1803561

**Published:** 2026-09-30

**Authors:** Ku Nurul Izzah Ku Seman, Shalisah Sharip, Idayu Badilla Idris, Syahnaz Mohd Hashim, Azmawati Mohammed Nawi

**Affiliations:** 1 Department of Psychiatry, Faculty of Medicine, Universiti Kebangsaan Malaysia, Kuala Lumpur, Malaysia; 2 Department of Psychology, AbdulHamid AbuSulayman Kulliyyah of Islamic Revealed Knowledge and Human Sciences (AHAS KIRKHS), International Islamic University Malaysia, Kuala Lumpur, Malaysia; 3 Department of Public Health Medicine, Faculty of Medicine, Universiti Kebangsaan Malaysia, Kuala Lumpur, Malaysia; 4 Department of Family Medicine, Faculty of Medicine, Universiti Kebangsaan Malaysia, Kuala Lumpur, Malaysia

**Keywords:** confirmatory factor analysis, cut-off point, quality of life, questionnaire, receiver operating characteristics

## Abstract

**Introduction:**

Validated quality of life assessments that show strong structural validity and screening utility with a cut-off point for psychiatric patients remain scarce. The initial validation of the New Psychological Quality of Life (PsychQOL) revealed a five-factor model through Exploratory Factor Analysis (EFA).

**Aim:**

This study aimed to assess the construct validity of the New Psychological Quality of Life (PsychQOL) measure through Confirmatory Factor Analysis (CFA) and Receiver Operating Characteristic (ROC) curve analysis, and to establish the preliminary cut-off score that differentiates those with and without psychiatric disorders.

**Methods:**

A total of 571 participants (334 healthy adults and 237 psychiatric patients) were recruited face-to-face and via online platforms for this cross-sectional study. Participants provided demographic information and completed the PsychQOL questionnaire.

**Results and Discussion:**

CFA of the final model showed acceptable fit in both samples after two items were removed. Multigroup CFA was conducted to test measurement invariance. Configural and metric invariance were supported, while scalar invariance was not fully established. The final Cronbach’s α for the 22- item questionnaire was 0.95. The ROC curve analysis yielded the AUC value of 0.83, *p* < 0.001, 95% CI = 0.80–0.87, suggesting that the questionnaire demonstrated an acceptable ability to distinguish those with and without psychiatric disorders. Overall, PsychQOL exhibits good reliability and acceptable construct validity, along with a preliminary cut-off point for distinguishing psychiatric from non- psychiatric populations.

## 1 Introduction

Quality of life (QOL) is a key outcome in psychiatric care, offering insights into patients’ functioning and well-being, beyond symptom severity (Gigantesco and Giuliani, 2011). Self-reported QOL assessments, whether generic or disorder-specific, capture overlapping but distinct aspects of QOL, highlighting the variability of QOL definitions and frameworks(Ware and Sherbourne, 1992; The WHOQOL Group, 1998; Ferrans et al., 2005). This diversity underscores the need for rigorous psychometric validation to ensure that QOL assessments are both theoretically robust and clinically relevant (Jacobs and Krueger, 2015; Clark and Watson, 2016). However, most existing QOL assessments were developed to describe quality of life rather than to classify individuals according to clinically meaningful thresholds (Guyatt et al., 1993; The WHOQOL Group, 1998). As a result, evidence supporting their use as screening tools, particularly for detecting poor QOL in psychiatric populations, remains limited. This gap is especially important in psychiatric settings, where timely identification of poor QOL is crucial for intervention planning (Kennedy, 2008).

Evaluating structural validity is a foundational step in questionnaire validation, often assessed using confirmatory factor analysis (CFA). CFA provides evidence for the dimensionality of an assessment and informs the appropriate scoring structure of an assessment (Brown and Moore, 2012; Prudon, 2015). While CFA offers primary evidence for structural validity, convergent validity can be further supported by examining associations with established QOL assessments. In psychiatric settings, widely used assessments such as the Quality of Life Enjoyment and Satisfaction Questionnaire-Short Form (Q-LES-Q-SF), the abbreviated World Health Organization Quality of Life Scale (WHOQOL-BREF) and Short Form-36 (SF-36) have shown strong psychometric structure, including structural validity, and have been validated across psychiatric populations (Keller et al., 1998; Oliveira et al., 2016; Riendeau et al., 2018). It has previously been observed that adapting and validating a current instrument is typically more time- and cost-effective and allows for comparisons across studies and cultures. However creating a new questionnaire enables the incorporation of constructs specific to the population, thus enhancing content validity, cultural relevance, and clinical applicability. It also allows for the customization of the tool to fit the local clinical environment, improves interpretability and practicality for regular use, and may offer increased sensitivity in identifying clinically significant differences or changes. In addition, several QOL assessments developed specifically for the psychiatric populations such as the S-QoL and Modular System QoL (MSQoL) questionnaire have undergone validity testing and show promising psychometric properties (Pukrop et al., 2003; Boyer et al., 2022). However, despite strong psychometric qualities, there is limited evidence for their ability to discriminate between psychiatric from non-psychiatric populations based on QOL scores, highlighting an important gap to be addressed through the establishment of clinically meaningful cut-off scores that can accurately distinguish individuals with psychiatric conditions from those without.

The lack of clinically meaningful thresholds limits the utility of these QOL assessments, particularly in resource-constrained psychiatric healthcare systems such as those in Malaysia (Chua, 2020). This issue highlights the need to examine whether QOL assessments can discriminate between individuals with or without psychiatric diagnoses, an aspect of validity known as known-groups validity. Receiver operating characteristic (ROC) curve analysis is a useful tool used to assess this validity, enabling the identification of cut-off points by balancing sensitivity and specificity across thresholds (Linden, 2006; Kumar and Indrayan, 2011). To date, the use of cut-off points in generic QOL assessments has focused on screening for mental health issues in populations with medical conditions. Previously suggested cutoffs included ≥2 on the EQ-5D for depression and anxiety (Short et al., 2021) and ≤ 38 on the SF-36 Mental Component Summary for identifying depression and anxiety risk in rheumatoid arthritis patients (sensitivity = 87.5%, specificity = 80.3%) (Matcham et al., 2016). Although these cut-off points are useful within medically ill samples, they are less applicable for distinguishing psychiatric from non-psychiatric populations in broader contexts without underlying medical conditions.

Cultural context plays a critical role in shaping individuals’ perceptions and evaluations of QOL (Skevington et al., 2020; Mao et al., 2021). Although Malay translations of QOL assessments such as WHOQOL-BREF, SF-36 and Q-LES-Q SF are available (Hasanah et al., 2003; Sararaks et al., 2004; Ibrahim et al., 2021), these instruments were developed in Western contexts, and may underrepresent areas that are essential in Malaysian cultures such as spirituality (Abdullah et al., 2018). To address this concern, the Psychological Quality of Life assessment (PsychQOL) was developed to fill the gap in culturally sensitive QOL assessment tailored for the Malaysian population. This assessment has undergone initial testing in the Malaysian population for face validity, as well as content and construct validity (Ku Seman et al., 2026). The earlier validation study identified five domains through exploratory factor analysis (EFA): Emotional Resilience, Religiosity and Values, Depression and Anxiety, Social Support and Vigor with factor loadings ranging from 0.41 to 0.87 in both healthy and psychiatric samples. Internal consistency was excellent for both healthy adults (Cronbach’s α = 0.91) and psychiatric patients (Cronbach’s α = 0.93).

While the assessment demonstrates initial psychometric strength, further validation is needed to determine the practicality and improve the measurement precision of the PsychQOL. Thus, this study aimed (i) to verify the latent structure using CFA, (ii) to examine convergent validity through correlations with the WHOQOL-BREF and (iii) to assess the known-groups validity of the PsychQOL through ROC curve analysis distinguishing those with and without psychiatric disorders. In addition, we explored a preliminary cut-off score associated with psychiatric status in this sample. By employing both CFA and ROC, we strengthened evidence for the theoretical validity and discriminant ability of the PsychQOL, offering preliminary evidence of the PsychQOL’s performance and strengthening the evidence base for psychological QOL assessment in psychiatric research.

## 2 Methods

### 2.1 Design, procedure and participants

This cross-sectional study was conducted from May 2024 to October 2024. Two groups of participants were recruited: healthy adults and psychiatric patients. Participants were recruited through various online platforms, such as Facebook, WhatsApp and Instagram, as well as through face-to-face recruitment. Of 339 healthy adults, 243 were recruited online, while 54 of 241 psychiatric patients were recruited online. Participants were recruited as a single large sample, which was then split into smaller datasets for separate analyses.

Virtual invitations containing a link to a Google Forms survey outlining the study objectives, procedures and questionnaire were posted across online platforms. Several non-governmental organizations focusing on advocacy for psychiatric patients were contacted to disseminate the invitation to their members to obtain a larger sample. Informed consent was obtained in the first section of the online survey form, while for face-to-face participants, information about the study was provided first and they then gave informed consent to participate. Face-to-face recruitment was conducted at the outpatient Psychiatric Clinic of Hospital Canselor Tuanku Muhriz, Malaysia. The questionnaires were self-administered on paper. All participants were asked to provide basic sociodemographic details and answer all the questions in the questionnaire. This study was approved by the Ethics Committee of the Faculty of Medicine, Hospital Canselor Tuanku Muhriz.

The inclusion criteria for the healthy adult group were Malaysian adults aged 18 and above, with no psychiatric disorder, and able to comprehend the questions in Malay language. Individuals with severe cognitive impairments were excluded from the study. For the psychiatric patient group, the inclusion criteria were Malaysian adults aged 18 and above, diagnosed by psychiatrists as having psychiatric disorder based on DSM-5 and documented in clinical notes, clinically stable and able to comprehend the questions. The status of psychiatric patients recruited online was determined through self-disclosure. The exclusion criteria were patients who were not clinically stable, for example, those with active psychosis, suicidal ideation, substance intoxication, or severe mood episodes.

### 2.2 Measures

#### 2.2.1 Psychological quality of life (PsychQOL) questionnaire

The PsychQOL is a brief 24-item questionnaire on psychological quality of life in Malay language. Findings from previous EFA showed that there were five factors identified, which were Vigor (three items), Emotional Resilience (seven items), Religiosity and Valued Living (five items), Depression and Anxiety (seven items), and Social Support (two items) (Ku Seman et al., 2026). The responses are recorded on 7-point scales, ranging from 1 for “Strongly Disagree” to 7 for “Strongly Agree.” In the final version of the 22-item questionnaire, six negative items (Q6, Q8, Q9, Q10, Q12 and Q16) from the Depression and Anxiety domain were reverse-scored before the total score was computed. The total score ranges from 22 to 154.

#### 2.2.2 World Health Organization quality of life-brief version (WHOQOL-BREF)

The WHOQOL-BREF is a 26-item questionnaire that focuses on QOL in four domains: physical, psychological, social relationships and environment (The WHOQOL Group, 1998). Responses are recorded on 5-point scales, ranging from 1 to 5, using a two-week recall period. The original version was developed in English, as an abbreviated form of WHOQOL-100 (The WHOQOL Group, 1998). The Malay adaptation has demonstrated satisfactory psychometric properties with moderate to high internal consistency across domains (α = 0.64 to 0.80) (Hasanah et al., 2003).

### 2.3 Statistical analysis

Statistical analysis was conducted using IBM SPSS version 26 software and IBM SPSS Amos version 29. Descriptive statistics were used to summarize the sociodemographic characteristics of the sample. The data met the assumption of univariate normality but did not satisfy multivariate normality. Domain and total scores are presented as means and standard deviations. In terms of reliability, internal consistency was evaluated using both Cronbach’s α and McDonald’s ω. Omega was calculated from the standardized factor loadings obtained in the CFA. Bland and Altman (1997) suggested that coefficient of 0.7 and above indicates acceptable reliability.

In the first part of the study, CFA was carried out using IBM SPSS AMOS Version 29, to assess the PsychQOL construct as explored through EFA conducted in a previous study (Ku Seman et al., 2026) with psychiatric samples. We tested the fit of the structure in the second study samples separately through CFA, followed by a multigroup CFA (MCFA) combining the study samples to verify the presumed factor structure within a set of observed variables in the two sample groups (Prudon, 2015). Hair et al. (2019) suggested that ordinal responses with at least four categories can be treated as continuous. Thus, using Maximum Likelihood Estimation, and Bollen-Stine bootstrap to address non-normality, the initial fit of the five-factor model (Emotional Resilience, Religiosity and Values, Depression and Anxiety, Social Support, and Vigor) was examined based on the chi-square (χ^2^) goodness-of-fit. However, due to the sample size and criticism of the suitability of the chi-square statistics for estimating fit, other indices of goodness-of-fit were also examined for acceptability of the fit (Prudon, 2015): Root Mean Square Error of Approximation (RMSEA) ≤ 0.08, Standardized Root Mean Square Residual (SRMR) ≤ 0.08, Tucker-Lewis Index (TLI) ≥ 0.90, Comparative Fit Index (CFI) ≥ 0.90 (Bentler and Bonett, 1980; Browne and Cudeck, 1992; Hu and Bentler, 1999). Any item with poor factor loadings below 0.50 was removed (Hair et al., 2019).

Using MCFA, three levels of measurement invariance were tested: configural, metric, and scalar measurement invariance. Establishing measurement invariance is very important because it provides evidence that the factor structure, factor loadings, and item functioning are comparable across populations, allowing for meaningful comparisons of PsychQOL scores between psychiatric and healthy adults samples. Configural invariance tests whether the same five-factor structure of PsychQOL can be applied to both sample groups and serves as the baseline model of MCFA. Metric invariance constrains the factor loadings and tests whether the questionnaire items relate to the domains similarly across groups, while scalar invariance tests the intercepts of the questionnaire items across groups (Putnick and Bornstein, 2016; Hair et al., 2019). MI was assessed using the likelihood ratio chi-square difference test; however, since this statistic tends to detect trivial parameter differences as statistically significant in large samples, an additional criterion-changes in CFI (ΔCFI) of less than 0.01 between nested models was used to support invariance (Cheung and Rensvold, 2002). Other criteria included changes in RMSEA (ΔRMSEA) of less than 0.015 and changes in SRMR (ΔSRMR) of less than 0.030 and 0.010 to support metric and scalar invariance, respectively(Chen, 2007). Where full invariance was not supported, partial invariance was examined using modification indices to identify non-invariant intercepts. This approach was undertaken because full scalar invariance was not achieved, indicating that some item intercepts differed between the psychiatric and healthy adult samples. Identifying and freeing these non-invariant intercepts allowed the model to achieve partial invariance while retaining most equality constraints (Putnick and Bornstein, 2016). Establishing partial invariance is important because it permits meaningful comparisons of latent factor scores across groups, despite minor differences in the functioning of specific items, thereby improving the validity of cross-group comparisons. Partial invariance was supported when the pattern of fit criteria (ΔCFI, ΔRMSEA, ΔSRMR) indicated acceptable change, even if the chi-square difference test remained significant. Noninvariant items were retained but flagged, and subsequent group comparisons involving them were interpreted with caution.

Subsequently, the convergent and divergent validity of the PsychQOL were assessed separately according to sample group to analyze whether the underlying factors were related as they expected in the theoretical construct. Conducting these analyses separately for each sample also allowed the validity of the PsychQOL to be evaluated independently within each group, ensuring that the instrument performs consistently across different populations. This was evaluated using composite reliability (CR) and average variance extracted (AVE) for convergent and discriminant validity. The calculations for CR and AVE followed Fornell and Larcker (1981). Moreover, Fornell and Larcker (1981) suggested that AVE should be 0.5 or above for convergent validity to be established, while for discriminant validity, the AVE of the factor must be greater than the squared correlation between factors. A CR of 0.70 is recommended by Hair et al. (2019) for internal consistency. In addition, addressing the second objective, the second part of the study focused on assessing convergent validity by examining the Pearson’s correlations between the PsychQOL total score and domains and the WHOQOL-BREF domain scores.

Next, the known-groups validity of the PsychQOL was evaluated using ROC curve analysis to assess the instrument’s ability to discriminate participants classified as psychiatric and non-psychiatric in this sample. Area Under the Curve (AUC) values ranged from 0.5 to 1.0, with values above 0.8 considered acceptable and useful (Muller et al., 2005; Çorbacıoğlu and Aksel, 2023). ROC analysis also enabled the identification of the optimal cut-off score that maximized the balance between sensitivity and specificity, thereby supporting the questionnaire’s potential use as a screening instrument. The cut-off point was chosen based on the highest Youden’s Index, defined as the point on the ROC curve with the maximum combined sensitivity and specificity (Hassanzad and Hajian-Tilaki, 2024). The sensitivity of the PsychQOL refers to the probability of correctly identifying individuals with psychiatric disorders, while specificity refers to the probability of the PsychQOL correctly identifying those without psychiatric disorders.

### 2.4 Transparency and openness

This study was not preregistered. The datasets, analytical materials, and other research materials supporting the findings of this study are available from the corresponding author upon reasonable request. Data was analysed using IBM SPSS Version 26 software and IBM SPSS Amos Version 29.

## 3 Results

### 3.1 Descriptive statistics

This study included 580 participants aged 18 to 70 years old (mean age = 35.5, SD = 11.2). Of the 580 participants, nine cases had missing data of one to two responses. Little’s MCAR test indicated that missingness was completely at random, χ^2^ (68) = 53.6, *p* = 0.90. Due to small percentage of missing data, listwise deletion was performed, leaving 571 participants (Li, 2013). There are 237 psychiatric patients and 334 healthy adults. Most of the participants were female (72.5%), and the highest ethnicity percentage was Malay (81.6%). 83.7% of the participants received a tertiary education. More than half of the participants were adults without any medical conditions (60.8%). Table 1 summarizes the participants’ demographic characteristics.

**Table 1:** Demographic details of the participants.

Participant characteristics	Frequency (%) or mean (SD)
Gender	
Male	157 (27.5)
Female	414 (72.5)
Age (years)	35.5 (11.2)
Ethnicity	
Malay	466 (81.6)
Chinese	63 (11.0)
Indian	20 (3.5)
Other ethnics	14 (2.4)
Not reported	8 (1.4)
Highest level of education	
Primary school	1 (0.2)
Secondary school	78 (13.7)
Certificate/Diploma	108 (18.9)
Bachelor’s degree	257 (45.0)
Master’s degree or higher	113 (19.8)
Others	14 (2.5)
Health condition	
With other medical condition	224 (39.2)
Without other medical conditions	347 (60.8)
Total score PsychQOL	
Psychiatric patients	90.09 (26.39)
Healthy adults	121.98 (19.21)

### 3.2 Confirmatory factor analysis

In this second study, a sequential analytical approach was adopted to evaluate the factor structure across two distinct datasets. The measurement model was first examined using CFA in the psychiatric sample, as the instrument was originally developed for clinical populations. Verifying the factor structure in a psychiatric cohort guaranteed that the suggested model accurately depicted the desired latent constructs in the specified clinical group prior to assessing its utility in a wider health population. Achieving an adequate model fit in the psychiatric sample demonstrates construct validity in the intended context and reduces the likelihood that any inadequate model fit seen in the health population results from a poorly defined measurement model instead of genuine differences in population traits. Thereafter, the identical CFA model was reassessed in the health population to determine the stability and generalizability of the factor structure across different groups. This step-by-step method allows researchers to assess if the tool retains its factorial validity outside psychiatric environments and reinforces its application in clinical and non-clinical groups.

The first CFA was conducted using the five-factor model from the previous validation study (Ku Seman et al., 2026) which is illustrated in Figure 1. This model has 24 observed variables, which are later classified into five latent variables: Emotional Resilience, Religiosity and Values, Depression and Anxiety, Social Support and Vigor. Item numbers in the figure match with the item number used in the questionnaire. Initial CFA with psychiatric samples showed poor fit, shown in Table 2. To improve the model fit, amendments were made on selected items. Modification indices (MI) indicated substantial residual covariance between Q20 and Q21 (MI = 55.567–37.649), suggesting item redundancy. Item Q20 from Religiosity and Values domain was therefore removed to improve model fit and reduce conceptual overlap between the two items. Item Q19 was also removed due to low factor loading of 0.41.

**Figure 1:**
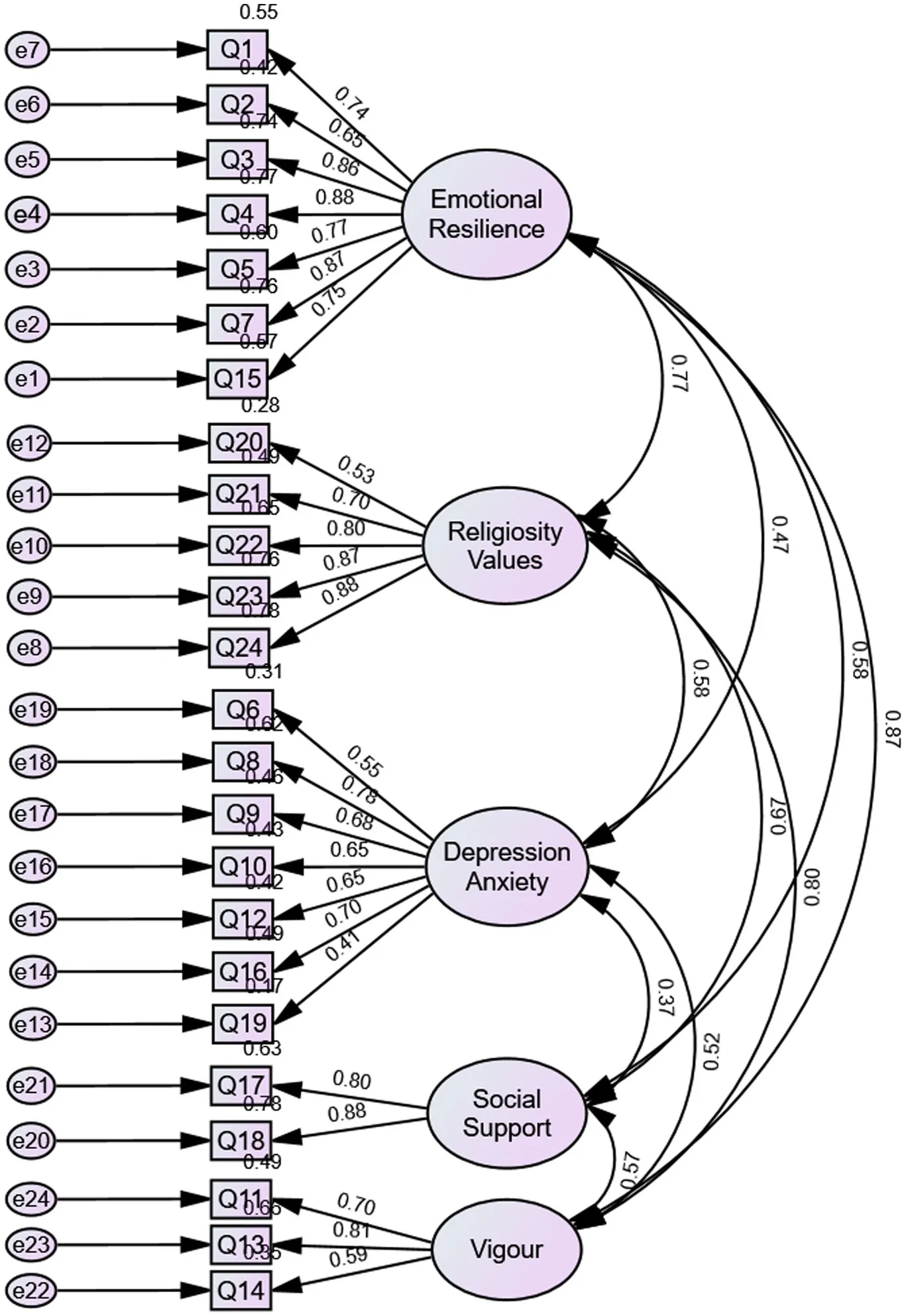
The initial 24-item model of the PsychQOL.

**Table 2:** Comparison of fit indices between the initial and the final models.

Index	Initial 24-item model	Final 22-item model	Final 22-item model
Sample	Psychiatric patients	Psychiatric patients	Healthy adults
Chi-square (df)	618.24 (242)	436.86 (199)	538.94 (199)
*p*-value	<0.001	<0.001	<0.001
*χ*^2^/DF	2.56	2.20	2.71
TLI	0.87	0.91	0.91
CFI	0.89	0.93	0.92
RMSEA	0.08	0.071	0.072
SRMR	0.06	0.05	0.05

The refined model of 22 items consists of five factors was analysed through second CFA using psychiatric samples. This second CFA was conducted to validate the revised measurement model after item refinement, confirm that the modified factor structure adequately fit the data, and ensure that the removal of items did not compromise the construct validity or psychometric properties of the instrument. Reassessing the refined model also provided evidence that the final version was stable, reliable, and suitable for subsequent validity analyses and clinical application. The findings revealed a slightly improved fit (shown in Table 2) compared to the initial model with indices within acceptable fit. The final model was illustrated in Figure 2 with factor loadings and correlations between domains. The correlation between the domain was 0.46 to 0.87 while factor loadings of the items were between 0.55 to 0.89. The CR ranges from 0.75 to 0.91 as illustrated in the Figure 2. This model structure was also tested using healthy adult samples, also revealing acceptable fit as summarized in Table 2. Evaluating the model in a healthy population provided evidence of its robustness and generalizability beyond psychiatric samples, indicating that the factor structure remained stable across different populations. This finding supports the broader applicability of the PsychQOL and suggests that the instrument measures the intended constructs consistently in both clinical and non-clinical groups. The factor loading ranges between 0.53 to 0.89, and the correlations between domains ranges from 0.50 to 0.81. The CR ranges from 0.71 to 0.90 as shown in Figure 3. This finding supports the broader applicability of the PsychQOL and suggests that the instrument measures the intended constructs consistently in both clinical and non-clinical groups. Subsequently, this 22-item model was tested in the pooled sample through MCFA. The five-factor model provided an acceptable fit for the pooled sample, χ^2^ (398) = 975.84, *p* < 0.001, RMSEA = 0.051 (90% CI = 0.047–0.055), CFI = 0.92, TLI = 0.91 and SRMR = 0.06. Furthermore, the metric model fit was also satisfactory, χ^2^ (415) = 998.37, *p* < 0.01, RMSEA = 0.050 (90% CI = 0.046–0.054), TLI = 0.91, CFI = 0.92 and SRMR = 0.05. The chi-square difference test between the metric and configural models was non-significant, Δχ^2^(17) = 22.53, *p* = 0.17. Other criteria were also below their respective threshold ΔCFI = 0.001, ΔSRMR = 0.002 and ΔRMSEA = 0.001. For scalar invariance, the chi-square difference test was significant, Δχ^2^(22) = 241.12, *p* < 0.001, ΔCFI = 0.030 exceeded the 0.010 threshold, and indicating a lack of full scalar invariance. However, ΔRMSEA = 0.007 and ΔSRMR = 0.000 both remained below the threshold. Table 3 summarized the details of invariance testing from configural to partial scalar models.

**Figure 2:**
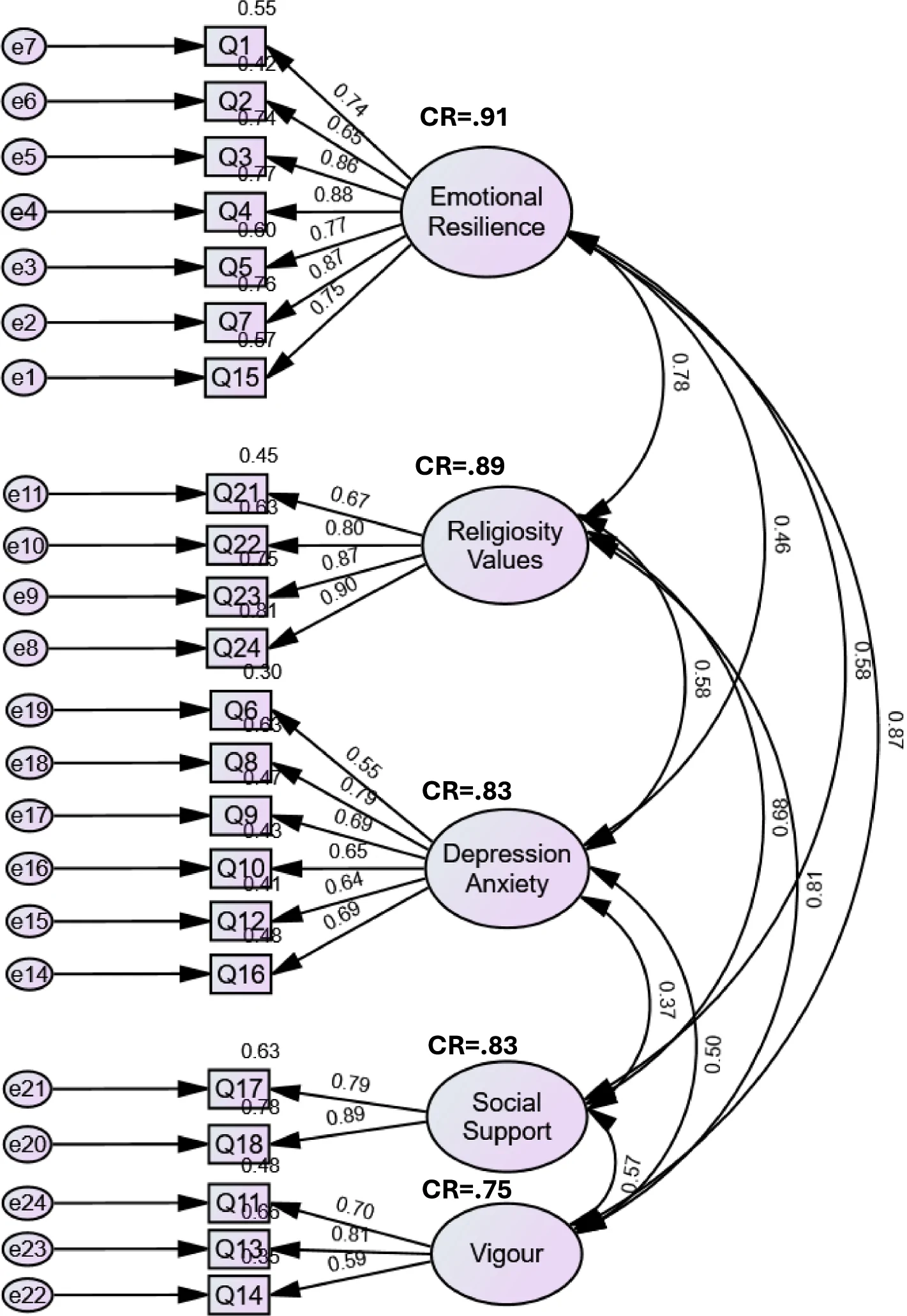
The final 22-item model of PsychQOL using psychiatric samples.

**Figure 3:**
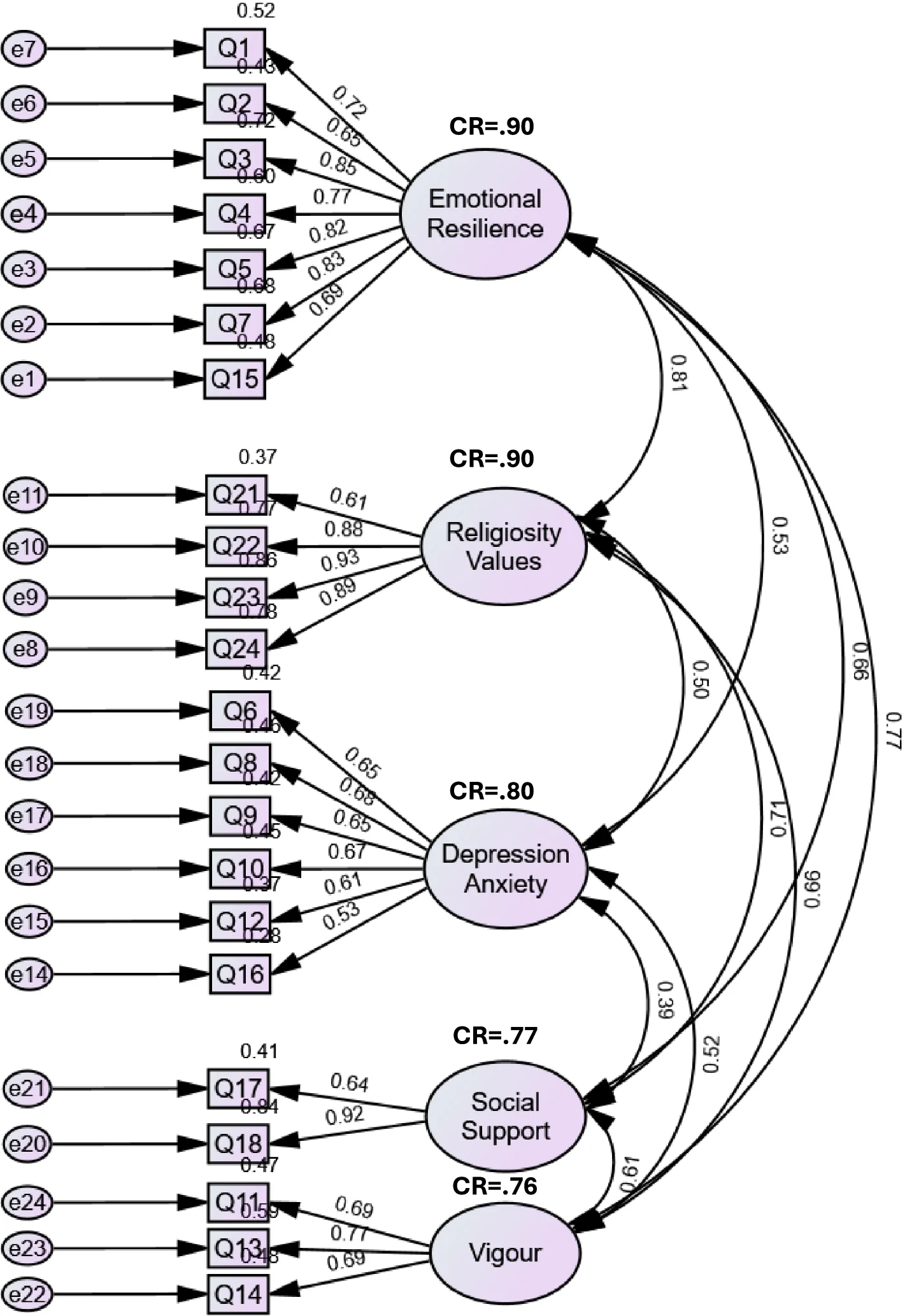
The final 22-item model of PsychQOL using healthy adults samples.

**Table 3:** Summary of measurement invariance testing across psychiatric and healthy adults samples.

Model	*χ* ^2^	df	CFI	TLI	RMSEA (90% CI)	SRMR	Δχ^2^	Δdf	*p*	ΔCFI	ΔRMSEA	ΔSRMR
Configural	975.84	398	0.921	0.908	0.051 (0.047–0.055)	0.0548	–	–	–	–	–	–
Metric	998.37	415	0.921	0.908	0.050 (0.046–0.054)	0.0533	22.53	17	0.165	0.001	0.001	0.0015
Scalar	1239.48	437	0.890	0.884	0.057 (0.053–0.061)	0.0533	241.12	22	<0.001	0.030	0.007	0.0000
Partial scalar (Q14, Q16 freed)	1218.46	435	0.893	0.886	0.056 (0.053–0.060)	0.0534	220.09	20	<0.001	0.027	0.006	0.0001

Due to these discrepancies, partial invariance was examined using modification indices to identify non-invariant intercepts. Items Q14 (Modification Indices = 4.39) and Q16 (Modification Indices = 5.71) were identified as the largest contributors causing the misfit. After freeing the Q14 and Q16 intercepts, the chi-square difference test relative to the metric model remained statistically significant, Δχ^2^(20) = 220.09, *p* < 0.001, and ΔCFI = 0.027 continued to exceed the 0.010 threshold. However, both ΔRMSEA = 0.006 and ΔSRMR = 0.000 remained within acceptable limits. These findings indicate that scalar invariance was not fully established. Residual intercept non-invariance in Q14 and Q16 suggests these items function differently across psychiatric and healthy adults groups, with has implications for direct score comparisons between groups (Cheung and Rensvold, 2002; Chen, 2007).

Moreover, for psychiatric samples, convergent validity was partially supported as both Vigor, and Depression and Anxiety have AVE values of 0.50 and below, while the other three domains exceeded the recommended threshold. Table 4 details the AVE of each factor and the squared correlation between domains for convergent and discriminant validity analysis (psychiatric samples) while the CR of each domain can be referred to Figure 2. As shown in Table 4, Depression and Anxiety, and Social Support satisfied the Fornell-Larcker criterion against all other domains. Religiosity and Values narrowly satisfied the criterion against Vigor. However, discriminant validity was not established between Emotional Resilience and Vigor (*r*^2^ = 0.76, exceeding Emotional Resilience’s AVE of 0.64), nor between Religiosity and Values, and Vigor or Emotional Resilience and Vigor (*r*^2^ of 0.65 and 0.76, respectively, both exceeding Vigor’s AVE of 0.50).

**Table 4:** Correlation coefficients squared and average for each factor (in brackets) for psychiatric samples.

Factor	Emotional resilience	Religiosity and values	Depression and anxiety	Social support	Vigor
Emotional resilience	(0.64)				
Religiosity and values	0.61	(0.66)			
Depression and anxiety	0.21	0.33	(0.45)		
Social support	0.34	0.46	0.14	(0.71)	
Vigor	0.76	0.65	0.26	0.33	(0.50)

For healthy adult samples, convergent validity was also partially supported as Depression and Anxiety had an AVE of 0.40 while Vigor was barely above the threshold (AVE = 0.52). As shown in Table 5, only three domains met the criterion for discriminant validity which were Religiosity and Values, Depression and Anxiety, and Social Support. Discriminant validity was not established between Emotional Resilience and Religiosity and Values (*r*^2^ = 0.65, exceeding Emotional Resilience’s AVE of 0.60), nor between Emotional Resilience and Vigor (*r*^2^ = 0.60, equal to Emotional Resilience’s AVE of 0.60 and exceeding Vigor’s AVE of 0.52).

**Table 5:** Correlation coefficients squared and average for each factor (in brackets) for healthy adults samples.

Factor	Emotional resilience	Religiosity and values	Depression and anxiety	Social support	Vigor
Emotional resilience	(0.60)				
Religiosity and values	0.65	(0.70)			
Depression and anxiety	0.28	0.25	(0.40)		
Social support	0.44	0.51	0.15	(0.63)	
Vigor	0.60	0.44	0.27	0.37	(0.52)

### 3.3 Internal consistency

For the 22-item questionnaire, Cronbach’s α was 0.95 and McDonald’s ω was 0.95 for the whole sample. Cronbach’s α for the domains was as follows: Emotional Resilience (α = 0.94), Religiosity and Values (α = 0.92), Depression and Anxiety (α = 0.85), Social Support (α = 0.80) and Vigor (α = 0.80). McDonald’s ω for the domains was as follows: Emotional Resilience (ω = 0.92), Religiosity and Values (ω = 0.89), Depression and Anxiety (ω = 0.83), Social Support (ω = 0.83) and Vigor (ω = 0.75).

### 3.4 Convergent validity with WHOQOL-BREF

For the second part of the study, we included only the subset of participants from the total sample who completed both the PsychQOL and the WHOQOL-BREF, as not all participants returned both questionnaires with complete responses. There are 297 participants: 242 healthy adults and 55 psychiatric patients. Convergent validity of the PsychQOL was examined using the correlations of the PsychQOL total score and domains with the WHOQOL-BREF domains. All correlations were highly significant, ranging from 0.52 to 0.86. The total score of the PsychQOL correlated strongly with the WHOQOL-BREF domains: Physical health (*r* = 0.79, *p* ≤ 0.001), Psychological (*r* = 0.86, *p* < 0.001), Social relationships (*r* = 0.71, *p* < 0.001) and Environment (*r* = 0.69, *p* < 0.001). Psychological functioning domain showed strongest correlation with WHOQOL-BREF Psychological domain (*r* = 0.85, *p* < 0.001). Depression and Anxiety domain (*r* = 0.68, *p* < 0.001) also shown highest correlation with the WHOQOL-BREF Psychological domain. Details regarding the correlations of the PsychQOL and the WHOQOL-BREF were shown in Table 6.

**Table 6:** Correlation between the PsychQOL domains and the WHOQOL-BREF domains.

Scale/Domain	WHOQOL physical health	WHOQOL psychological	WHOQOL social relationship	WHOQOL environment
PsychQOL total scale score	0.79**	0.86**	0.73**	0.70**
PsychQOL domain 1: emotional resilience	0.73**	0.76**	0.63**	0.63**
PsychQOL domain 2: religiosity and values	0.65**	0.79**	0.66**	0.63**
PsychQOL domain 3: depression and anxiety	0.60**	0.68**	0.56**	0.52**
PsychQOL domain 4: social support	0.57**	0.61**	0.65**	0.59**
PsychQOL domain 5: Vigor	0.79**	0.77**	0.65**	0.62**

**Correlation is significant at 0.01 level (2-tailed).

### 3.5 Discrimination between groups and cut-off values determination

Next, for the third part of the study involving ROC analysis, we included all samples (N = 571) differentiated by the psychiatric status. The ROC curve for the PsychQOL scores of the 22-item version is shown in Figure 4. The ROC curve revealed AUC value of 0.83, *p* < 0.001, 95% CI = 0.80–0.87, suggesting that the questionnaire showed acceptable ability to distinguish those with and without psychiatric disorders, contributing to known-group validity. A cut-off point of 105 was identified based on the highest Youden’s Index value (J = 0.58, 95% CI = 0.52–0.65), with a sensitivity of 0.75 (95% CI = 0.69–0.80) and specificity of 0.84 (95% CI = 0.79–0.87).

**Figure 4:**
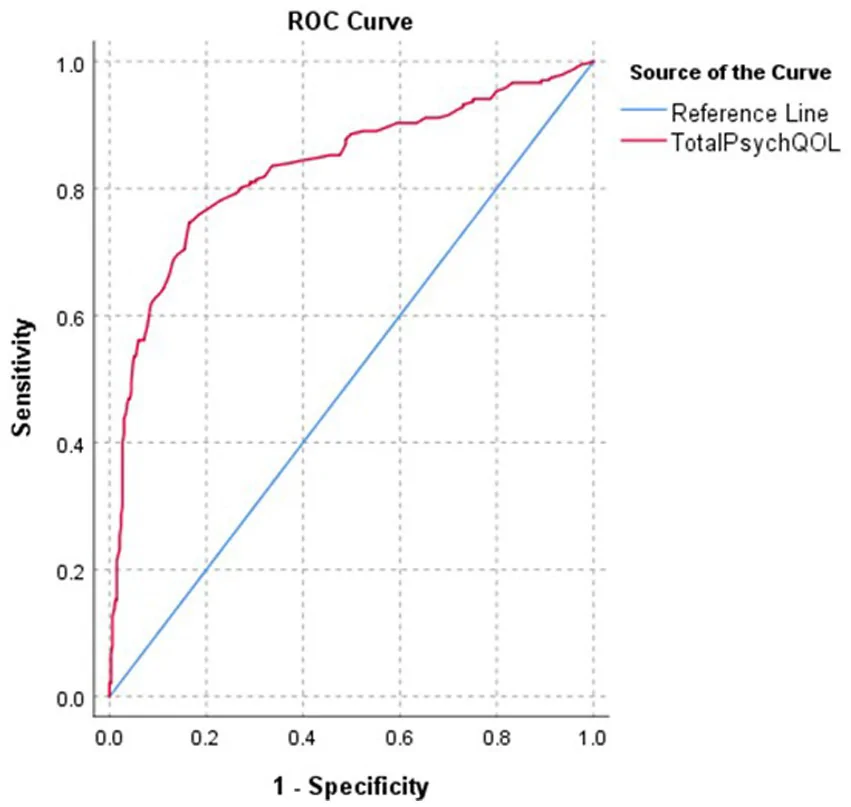
ROC curve for the PsychQOL total score.

## 4 Discussion

In this present study, the validation of the psychological quality of life instrument, the PsychQOL was further analysed, expanding the early validation study of the PsychQOL (Ku Seman et al., 2026). The five-factor structure remained stable following the removal of two items, reducing the initial 24-item pool to 22 items without collapsing or merging domains, indicating that the removed items were not essential to the retained factor structure, even if this structural stability does not establish the instrument’s overall construct validity. The final 22-item model showed adequate fit in both the psychiatric and healthy adults samples, supporting broader applicability of the PsychQOL and suggests that the instrument measures the intended constructs consistently in both clinical and non-clinical groups, lending partial support to its construct validity. Internal consistency for all five domains was excellent (Cronbach’s α and McDonald’s ω), indicating that the final model has not sacrificed reliability for parsimony, an important consideration for a scale intended for practical, repeated administration in clinical and research settings (Sharma, 2022). Convergent and discriminant validity, however, were only partially established in both samples, tempering the construct validity evidence provided by model fit and reliability alone.

Two items from the initial model of 24 items were deleted in the process of improving the model. Item Q20 (When I faced health issue, I started getting closer to God) was removed instead of Q21 in the final model because its wording reflected QOL less, as it focused more on the act of seeking spiritual coping. Spiritual coping is known to be associated with QOL, however, its role has been suggested as a mediator (Surzykiewicz et al., 2022; Okwuosa et al., 2023), rather than as a QOL outcome, which is incongruent with how other items in the PsychQOL were framed. Next, Item Q19 (My sexual desire was disturbed) had low factor loading. The association between decreased sexual desire and depression has long been documented (Sabic et al., 2021; Lee et al., 2024). Despite that relationship, the item loading was low, suggesting that sexual desire may not be a strong direct indicator of psychological quality of life. One possible explanation is that sexual desire is a multidimensional construct influenced by biological, psychological, interpersonal, cultural, and treatment-related factors rather than psychological well-being alone(Malary et al., 2015). In psychiatric populations, sexual desire may also be affected by psychotropic medication, relationship status, and age, which can weaken its association with the underlying psychological QOL construct (Park et al., 2015; Montejo et al., 2018; Kumari et al., 2024). Furthermore, decreased sexual desire is often considered a component of sexual dysfunction that primarily influences quality of life through its effect on sexual satisfaction rather than directly (Chao et al., 2011; Eissa et al., 2022).

Convergent and discriminant validity, however, were only partially established in both samples, tempering the construct validity evidence provided by model fit and reliability alone. Although the PsychQOL demonstrated an acceptable factor structure and good internal consistency, only partial evidence of convergent and discriminant validity was obtained. This suggests that while the questionnaire is structurally sound and reliable, some constructs may not be measured as distinctly or consistently as intended. Therefore, the evidence supporting its construct validity is encouraging but not definitive, indicating that further refinement and validation of certain factors may be required. The Depression and Anxiety domain showed poor AVE consistently across both samples, indicating that the latent construct explained less than half of the variance in its indicators (Fornell and Larcker, 1981). In addition to measurement error, this finding may reflect the multidimensional nature of depression and anxiety, which comprise related but distinct symptom clusters that do not necessarily converge on a single latent factor (Vares et al., 2015). The broad content of the domain, together with variability in symptom presentation across respondents and potential overlap with other psychological constructs, may have further reduced the shared variance among items. Consequently, although the items exhibited acceptable internal consistency, they may not capture the underlying construct as cohesively as intended. Thus domain scores for this domain should be interpreted cautiously and its item set considered for refinement in future revisions.

Similarly, discriminant validity was partially established. Elevated shared variances among several domain pairs suggest conceptual overlap, as one domain may contribute disproportionately to the variance in another, complicating the interpretability of individual domains. Only Religiosity and Values, Depression and Anxiety, and Social Support demonstrated discriminant validity, indicating that for these three domains, variance in their items was better explained by their own domain than by overlap with other domains. Examining the pattern of high correlations and shared variances, Emotional Resilience and Vigor showed a consistently high correlation across sample groups. This is consistent with a previous study documenting differing perceptions of psychological QOL, where some items were considered to belong to different domains depending on the sample (Ku Seman et al., 2026). In the earlier EFA using healthy adults as samples, three items that belong to the current Emotional Resilience were previously under Vigor. The three items relate to the quality of completing tasks, which relates to self-efficacy, and has been proposed to influence resilience in adults (Pahwa and Studies, 2022), although an alternative explanation is that they instead reflect physical wellbeing (Capio et al., 2023). Furthermore, religiosity and life values, under the umbrella of spirituality, had often correlated with psychological well-being (Leung and Pong, 2021; Shakerian et al., 2021). Individuals with poorer spiritual wellbeing have been reported to experience higher levels of stress, depression, and anxiety symptoms. Emotional regulation has been proposed to mediate the relationship between spirituality and psychological wellbeing, highlighting the interplay between these two constructs (Graça and Brandão, 2024), which may explain the high correlation between the Religiosity and Values domain and the Emotional Resilience domain.

Furthermore, measurement invariance was tested using MCFA to evaluate whether the model is equivalent in both sample groups. Configural and metric invariance were established, suggesting that this five-domain structure holds in psychiatric and healthy adults samples, and the questionnaire items relate to their respective domains similarly across groups (Putnick and Bornstein, 2016). However, full scalar invariance was not established, indicating that participants in each group did not always endorse items identically even at equivalent underlying levels of psychological QOL. This is not an uncommon finding when comparing clinical and non-clinical populations, as psychiatric patients may interpret certain items through the lens of their symptom experience, while non-psychiatric respondents may draw on different reference points when responding to the same content (Schuler et al., 2014). One source item is Q14 which addresses sleep quality. Given the high likelihood of sleep disturbance in psychiatric disorders, this item would be expected to show systematically lower endorsement among psychiatric patients, independent of their underlying QOL level (Freeman et al., 2020). Similarly, another source item, Q16 focuses on loneliness (reverse-scored item where higher endorsement reflects greater loneliness). Higher endorsement of the item was expected as psychiatric patients often experienced stigma-related reductions in social network size and participation (Adu et al., 2023; Caple et al., 2023), which are associated with increased loneliness in psychiatric populations (Meltzer et al., 2012). This could contribute to how the items behaved differently in the dataset, contributing to non-invariance at intercept level.

In terms of the external convergent validity of the PsychQOL domains with the WHOQOL-BREF domains, the strong and statistically significant correlations suggest that the domains are related, supporting the interpretation that both assessments measure related aspects of the QOL construct. Both domains were related closely to the WHOQOL-BREF Psychological domain, which is conceptually consistent with expectations. The WHOQOL-BREF Psychological domain includes items related negative emotions, positive affects, self-esteem and spirituality, which align closely with the PsychQOL domains (The WHOQOL Group, 1998). Besides that, weaker correlations of the PsychQOL domains with WHOQOL-BREF Physical, Social and Environment domains were observed. This pattern is theoretically coherent, given that the PsychQOL was designed to focus on psychological aspects of QOL rather than physical health, social relationship and environmental resources. The differentiated pattern of associations indicates that the PsychQOL measures psychological QOL constructs that are related to, but not fully overlapping with non-psychological QOL domain.

In addition, the PsychQOL demonstrated adequate discriminatory ability for screening between those with and without psychiatric disorders at a proposed cut-off score of 105. Lower PsychQOL scores were strongly associated with membership in the psychiatric group, supporting the assessment’s known-groups validity. At this threshold, sensitivity was 75% and specificity was 84%, reflecting a favorable balance between correctly identifying individuals in the psychiatric group and minimizing misclassification of individuals in the healthy adults group. Accordingly, the preliminary cut-off score provides early evidence to support screening for group membership. In future validation studies, the potential application of PsychQOL as a guide for referral to targeted psychological intervention should be assessed. Furthermore, the high specificity of the PsychQOL facilitates efficient use of clinical resources, reducing false positives and unnecessary referrals among healthy adults. Although the identified cut-off demonstrated acceptable discriminatory performance in the present sample, it was both derived and evaluated using the same dataset. Therefore, the cut-off should be regarded as provisional and requires cross-validation in independent samples before it can be recommended for broader clinical application.

Some limitations have been identified in this study. First, in the domain-level psychometric performance discussed above, the Emotional Resilience and Vigor domains showed insufficient discriminant validity, and the Depression and Anxiety domain showed low AVE in both samples. These findings suggest some PsychQOL domains may benefit from item refinement or re-examination of domain boundaries in future revisions, and scores from the affected domains should be interpreted with corresponding caution. Second, the preliminary cutoff point was derived from a sample of mixed psychiatric diagnoses and healthy adults. The cut-off point separating those with and without psychiatric disorders may differ if the composition of the psychiatric diagnosis changes. Thus, future research should attempt to replicate this in an independent sample with a specific diagnostic group or a balanced psychiatric composition, to improve the utility of the cut-off point. Future studies should also addressed test–retest reliability and longitudinal responsiveness are separate measurement properties and were not evaluated in this cross-sectional study.

Furthermore, the sample composition was predominantly female and Malay, as in the earlier study of PsychQOL (Ku Seman et al., 2026). Although the percentages of males and other racial groups increased in this study, the proportions were far from balanced. This imbalance limits the generalisability of the findings, as gender and cultural differences may influence the perception of QOL (Kagawa-Singer et al., 2010; Zhang et al., 2018), leaving uncertainty about the PsychQOL performance in a more heterogeneous population. Future studies could examine the performance of this questionnaire in a balanced dataset to increase the generalisability of the results to wider populations. It is also important to highlight that most of our healthy adults participants were recruited online while the psychiatric patients were mostly recruited through physical setting. This mixed-mode design may introduce mode effects due to the use of different questionnaire administration methods. Previously, using internet surveys has been associated with coverage bias as participation is influenced by access to technology and internet-enabled devices, potentially excluding individuals without such access from the study population (de Leeuw and Hox, 2018). This may influence the demographic of the respondents, as internet surveys have been shown to recruit more younger respondents compared to traditional methods (Börkan, 2010). This may impact the findings as perception of QOL vary with age (Lavalekar and Karmalkar, 2017), potentially contributing to differences in responses and factor structure stability. Lastly, the use of self-disclosed rather than clinically verified psychiatric diagnosis for online participants represents a methodological constraint when interpreting the results as preliminary evidence of known-groups validity.

In conclusion, the findings of this study support the previously proposed five-factor model in a larger, independent sample. The revised 22-item model demonstrates an acceptable fit indices and internal reliability. Measurement invariance was fully established in the configural and metric models, while only partial scalar invariance was achieved. We recommend an exploratory cut-off point of 105 with satisfactory sensitivity and specificity for differentiating psychiatric patients from healthy adults. This questionnaire serves as an alternative tool with adequate psychometric properties for assessing psychological QOL in the Malaysian population.

## Data Availability

The raw data supporting the conclusions of this article will be made available by the authors, without undue reservation.
